# Simultaneous Urea and Phosphate Recovery from Synthetic Urine by Electrochemical Stabilization

**DOI:** 10.3390/membranes13080699

**Published:** 2023-07-27

**Authors:** László Koók, Kristóf Bence Nagy, Ilona Nyirő-Kósa, Szilveszter Kovács, Jan Žitka, Miroslav Otmar, Péter Bakonyi, Nándor Nemestóthy, Katalin Bélafi-Bakó

**Affiliations:** 1 Research Group on Bioengineering, Membrane Technology and Energetics, University of Pannonia, Egyetem u. 10, H-8200 Veszprém, Hungary; 2 ELKH-PE Environmental Mineralogy Research Group, University of Pannonia, Egyetem u. 10, H-8200 Veszprém, Hungary; 3 Research Institute of Biomolecular and Chemical Engineering, Nanolab, University of Pannonia, Egyetem u. 10, H-8200 Veszprém, Hungary; 4 Institute of Macromolecular Chemistry, AS CR, Heyrovsky Sq. 2, 162 06 Prague, Czech Republic; 5 TailorMem, Zájezd 8, 273 43 Zájezd, Czech Republic

**Keywords:** electrochemical pH modulation, phosphate recovery, urea stabilization, urease inhibition, urine

## Abstract

Urine is a widely available renewable source of nitrogen and phosphorous. The nitrogen in urine is present in the form of urea, which is rapidly hydrolyzed to ammonia and carbonic acid by the urease enzymes occurring in nature. In order to efficiently recover urea, the inhibition of urease must be done, usually by increasing the pH value above 11. This method, however, usually is based on external chemical dosing, limiting the sustainability of the process. In this work, the simultaneous recovery of urea and phosphorous from synthetic urine was aimed at by means of electrochemical pH modulation. Electrochemical cells were constructed and used for urea stabilization from synthetic urine by the in situ formation of OH^-^ ions at the cathode. In addition, phosphorous precipitation with divalent cations (Ca^2+^, Mg^2+^) in the course of pH elevation was studied. Electrochemical cells equipped with commercial (Fumasep FKE) and developmental (PSEBS SU) cation exchange membranes (CEM) were used in this study to carry out urea stabilization and simultaneous P-recovery at an applied current density of 60 A m^−2^. The urea was successfully stabilized for a long time (more than 1 month at room temperature and nearly two months at 4 °C) at a pH of 11.5. In addition, >82% P-recovery could be achieved in the form of precipitate, which was identified as amorphous calcium magnesium phosphate (CMP) by using transmission electron microscopy (TEM).

## 1. Introduction

The rapidly increasing demand for food is nowadays challenged by the price and depletion of non-renewable-based sources and technologies of valuable phosphorous and nitrogen (fertilizer) production. Source-separated urine is known to be an inexhaustible source of N and P; however, its practical utilization technologies are currently limited by a line of aspects related to the special nature of urine. Most importantly, the instability of source-separated urine does not allow for the use of the conventional centralized treatment approach [1,2,3]. This unstable characteristic can be attributed to the naturally wide occurrence of positive microorganisms in urease, which are accountable for the relatively rapid degradation of urea to ammonia and bicarbonate according to Equation (1) [4,5]. This leads to the volatilization of NH_4_^+^, resulting in N-loss [6]. Moreover, significant emissions of odor and pollution hamper the efficacy of collection, transportation and storage processes [7].

As a further barrier to the practical realization of its utilization for resource recovery, the Ca^2+^ and Mg^2+^ ions present in source-separated urine can lead to the severe clogging of pipelines [6,8,9]. The precipitates of calcium and magnesium salts form during the natural pH increase in non-stabilized urine.

(1) $$\mathrm{CO}({\mathrm{NH}}_{2}{)}_{2}+2\;H_{2}O\;\to \;{\mathrm{NH}}_{3}\;+\;{\mathrm{NH}}_{4^{+}}\;+\;{\mathrm{HCO}}_{3^{-}}$$

Overall, it can be said that the traditional centralized scheme of collection–transportation–storage–treatment is likely not a feasible way to approach the resource recovery of source-separated urine [10]. Therefore, on-site treatment methods, including a variety of electrochemical techniques (such as electrochemical precipitation, stripping, oxidation or capacitive deionization), are widely investigated [2]. Among them, electrochemical systems for simultaneous N- and P-recovery are of utmost interest.

For example, Arve and Popat [11] have demonstrated an efficient electrochemical process, in which an oxidant, namely, hydrogen peroxide (H_2_O_2_), is produced at the cathode in situ in a two-chambered electrochemical cell. This way, the hydrogen peroxide could inactivate the urease enzyme, which leads to nearly 80% urea preserved and stabilized for at least 140 days. Moreover, they concluded that a higher current density and longer process time lead to more efficient stabilization [11]. This also shows the benefit of using electrochemically formed oxidants as biocidal agents leading to the easier storage of stabilized urea even at room temperature [12].

Capacitive deionization represents a technique using cation and anion exchange membranes, by which, in addition to phosphorous recovery from urine (in the form of phosphoric acid product), a desalted urea-rich solution can also be separately obtained [13]. However, the treatment does not accomplish urea stabilization, and challenges mainly related to the ion exchange membranes and their physico-chemical properties limit the technological implementation [13].

Electrochemical pH modulation (also known as electrohydromodulation) was shown previously to be a practical method for recovering nitrogen and phosphorous from municipal wastewater [14]. This approach can be adapted for the treatment of source-separated urine, and this has also been demonstrated by Paepe et al. [15]. The stabilization of source-separated urine by means of electrochemical pH modulation leads to the prevention of urea degradation (via urease enzyme inhibition), potentially allowing storage and/or collection without N-loss until further processing [14,15]. This can be achieved by cathodic OH^−^ production according to Equation (2), which, on the one hand, stabilizes the urea by the increase in pH above 10, and on the other hand, leads to the (partial) precipitation of phosphorous, mostly in the form of struvite, hydroxyapatite or other forms of Ca/Mg phosphates [16,17].

H_2_O + e^−^ → ½ H_2_ + OH^−^ (*E*^0^ = −0.8227 V vs. SHE) (2)

Meanwhile, the anodic reaction usually involves the oxidation of water, as described in Equation (3).

(3) $$H_{2}O\;\to \;O_{2}\;+\;2\;H^{+}\;+\;2\;e^{-}\;(E^{0}\;=\;1.229\;V\;\mathrm{vs}.\;\mathrm{SHE})$$

The so-obtained electrochemical half-cells are separated by an ion exchange membrane or a combination of mono- and/or bipolar membranes, sustaining a pH gradient between the electrolytes. The advantage of such an approach is that no external chemical addition is needed, unlike in the case of, e.g., the inhibition of enzymatic urea hydrolysis by increasing the pH using H_2_O_2_, or decreasing the pH with added acids [18,19]. Furthermore, the simultaneous phosphorous precipitation (i) allows the physically separated recovery of N and P, and (ii) sufficiently removes the bivalent cations, thus preventing scaling and clogging issues. In fact, P-recovery efficiency is determined by the available Ca^2+^ and Mg^2+^. The stabilized urea-rich solution can be stored and utilized as urea fertilizer, or utilized as a N-source, e.g., in ammonia recovery applications after hydrolysis. Phosphate-containing precipitates separated from the solution can be used as slow-release P-sources [20]. However, the formation of struvite from non-hydrolyzed urine is limited by the low amounts of ammonium and magnesium [9,21].

In this work, two-chamber electrochemical systems were investigated for simultaneous urea stabilization and phosphorous recovery by electrochemically induced pH modulation and phosphate precipitation. Reactor designs are tested with cation exchange membranes (CEM), by which the loss of P due to cross-membrane migration can be avoided. Two CEMs were used, namely, a commercial Fumasep FKE CEM as reference, and a developmental PSEBS SU CEM for performance comparison. The study aims to evaluate the stabilization process kinetics in order to estimate the necessary process time and amount of OH^−^ ions for achieving an intermediary catholyte pH of 11.5. The stability of the electrochemically pH-modulated catholyte was investigated over longer periods of time at room temperature and 4 °C in terms of changes in urea concentration over time. The efficiency of P-recovery and the composition of P-containing precipitate are also addressed in the presented work.

## 2. Materials and Methods

### 2.1. Membranes

Two homogeneous, non-reinforced CEM were used in the course of the experiments. Fumasep FKE CEM (FuMa-Tech, Bietigheim-Bissingen, Germany) was applied as a commercial reference material. Its swollen thickness was 66 ± 3 μm. PSEBS SU is a developmental CEM developed at the Czech Academy of Sciences. It is based on a functionalized polystyrene-block-poly(ethylene-ran-butylene)-block-polystyrene (PSEBS) polymer backbone. PSEBS SU samples with swollen thickness of 158 ± 12 μm were applied in the electrochemical cells. Both membranes were activated prior to use via acid–base treatment with 1 M HCl and 1 M NaOH. Further details on the membrane properties and activation can be found elsewhere [22].

### 2.2. Experimental Setup

**Figure 1:**
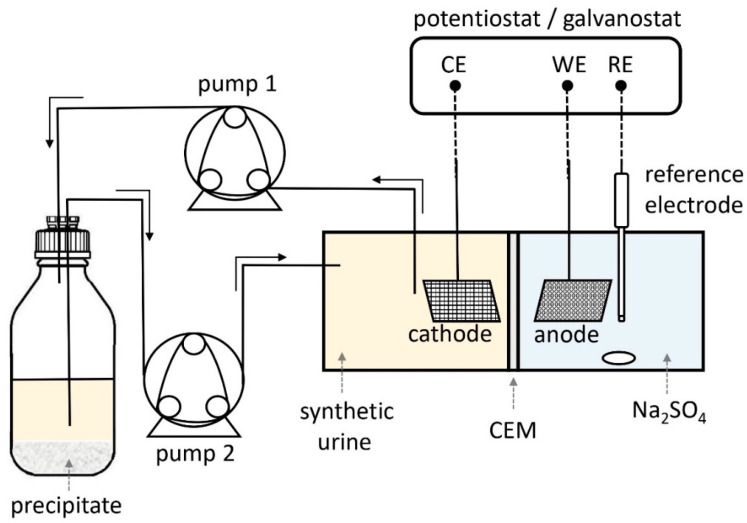
Experimental setup for electrochemical stabilization of synthetic urine.

The electrochemical cell used in this study was a cubic reactor with anolyte and catholyte working volumes of 200–200 mL. The half-cells were separated by a 2 cm × 2 cm CEM (either Fumasep FKE or PSEBS SU). The anode was a 2.5 cm × 3.5 cm Pt gauze electrode (BAS, Tokyo, Japan), while the cathode was a 3 cm × 3 cm stainless steel mesh, connected to a Ti wire (0.2 mm in diameter). In addition, a saturated Ag/AgCl reference electrode (SE11, Meinsberg Sensortechnik GmbH, Waldheim, Germany) was placed in the anode chamber for maintaining the galvanostatic control. A 250 mL glass bottle was connected to the cathode chamber by two peristaltic pumps and silicone tubing for the efficient circulation of the catholyte and to collect the forming precipitates (Figure 1). As the precipitation occurs at the cathode electrode, pumping the solution from the vicinity of the electrode directly moves the particles into the precipitate collection bottle, where they can settle on the bottom.

### 2.3. Elecrochemical Stabilization Process

The synthetic urine solution was made based on the composition recommended by Sarigul et al. [23] containing 15 g L^−1^ urea (99%, M = 60.06 g mol^−1^). The catholyte was made by the 10-fold dilution of the synthetic urine, and in the beginning of the experiments, 200 mL and 150 mL catholyte was filled into the cathode chamber and into the precipitate bottle, respectively. During the process, the catholyte was continuously recirculated with a 4 mL min^−1^ flow rate. The anolyte consisted of a 0.2 M Na_2_SO_4_ solution (≥99%, M = 142.04 g mol^−1^), which was stirred at 150 rpm using a magnetic stirrer. The role of the Na_2_SO_4_ solution in the electrochemical cell setup equipped with CEM is to sufficiently ensure cation transport across the membrane by providing Na^+^ ions. The concentration was selected to be high enough to avoid Na^+^ depletion during the pH modulation process (as the transport of H^+^ formed at the anode can be considered significantly lower compared to sodium ions in the present concentration range). The sulfuric acid forming in the anolyte can be further utilized, e.g., as a cleaning agent and for scaling removal in a source separation toilet [15]. The cathode, anode and Ag/AgCl electrodes were connected to the counter, working and reference electrode ports of a potentiostat/galvanostat (PamsSens 3, PalmSens, Houten, Netherlands). The constant current density was set to 60 A m^−2^ (relative to the membrane surface area) in the chronopotentiometric measurements. The experiments were carried out at room temperature (20–22 °C).

### 2.4. Analysis

The pH and conductivity (σ) of the electrolytes were measured with a PCE-228 pH meter (PCE Instruments, Meschede, Germany) and a WTW Cond 3110 conductivity sensor (Xylem-WTW, Burlington, MA, USA). The determination of urea was carried out via spectrophotometry using the 4-(dimethylamino)benzaldehyde method [24] and a Hach Lange DR 3900 spectrophotometer (Hach, Loveland, CO, USA). Samples taken from the catholyte and the stabilized solution were diluted 10-fold. The reagent solution consisted of (per 100 mL) 92 mL absolute ethanol (M = 46.08 g mol^−1^), 8 mL cc. HCl (M = 36.46 g mol^−1^) solution and 1.472 g 4-(dimethylamino)benzaldehyde (99%, M = 149.19 g mol^−1^). The diluted sample and the reagent solution were mixed at a 1:1 ratio. After 10 min, a yellow color developed, and the absorbance was determined at 440 nm.

The measurement of orthophosphate was also undertaken by spectrophotometry analysis using the α-ascorbic acid method. The (500-fold) diluted samples and the reagent solution were mixed in a 5:1 ratio. After 15 min, a blue color developed and the absorbance was determined at 882 nm [25].

The physical and chemical characterization of the precipitate was carried out using transmission electron microscopy (TEM). The solution containing the flake-like precipitate was filtered through a 0.45 μm MCE membrane filter using a vacuum pump. In order to avoid crystal formation from the solution during dehydration, we washed the precipitate with 20 mL of isopropanol. We carefully touched the membrane filter with a Cu grid covered with the continuous carbon film used in TEM examinations, dried the grid and analyzed the sample. TEM was performed using a ThermoFisher Talos F200X electron microscope (Thermo Fisher Scientific Inc., Waltham, MA, USA), operated at 200 kV accelerating voltage and equipped with a four-detector Super-X energy-dispersive X-ray spectroscopy (EDS) system for elemental analysis. Images were collected both in TEM (bright-field (BF) and high-resolution (HRTEM) images) and scanning (STEM) modes (high-angle annular dark-field, HAADF images). The elemental compositions of the particles were determined using EDS mapping in STEM mode. The choice of TEM for precipitate was based on the possible nanocrystalline structure of the samples (which could lead to the loss of peaks in other techniques such as X-ray dispersion measurements).

## 3. Results and Discussion

### 3.1. Electrochemical pH-Shift of Synthetic Urine

**Figure 2:**
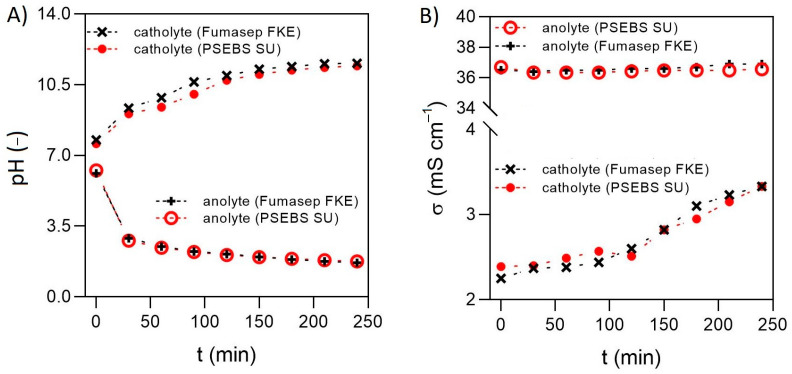
The electrolyte pH (**A**) and conductivity (**B**) as a function of time for Fumasep FKE and PSEBS SU membranes.

In order to avoid urea degradation and allow sufficient storage until further processing, an electrochemically induced pH increase was shown to be a promising technique. As key factors during the treatment, the final pH value and the operational time could be highlighted. In these experiments, 60 A m^−2^ was selected as the applied current density, based on previous experiences. As for the desired final pH of the solution, De Paepe et al. [15] underlined that the lower bound pH of 11—although being considered as high enough for urease enzyme inhibition [21]—does not allow efficient and long-term stabilization of urea. Therefore, in their work, a final pH of 12 was set in the experiments, by which they successfully obtained urea solutions that were stable for >18 months [15]. However, the additional electric energy requirement and time needed for increasing the pH above 11 can be comparable to those required for reaching pH = 11. Figure 2A shows the change in electrolyte pH values as a function of operational time, from which it can be seen that the rate of pH elevation in the catholyte significantly decreases above pH = 11, mostly due to the buffering effect of NH_4_^+^/NH_3_, HCO_3_^−^/CO_3_^2−^ and HPO_4_^2−^/PO_4_^3−^ systems [15]. Thus, in these experiments, an intermediate pH value of 11.5 was selected and evaluated for urea stabilization.

As can be seen in Figure 2A, the progress of pH curves gradually slowed down over time above pH ≈ 10.5. Nevertheless, the overall changes in catholyte and anolyte pH values were similar in both CEMs. The targeted pH = 11.5 of the catholyte was achieved after 4 h, under which the anolyte pH decreased to ~ pH = 1.7 in both cells. During the 4 h, an average conductivity increase of 1 mS cm^−1^ could be observed in the catholyte, while the anolyte σ showed a more steady value, with a 0.3–0.5 mS cm^−1^ overall increase (Figure 2B), regardless of the membrane type.

Based on the results, it can be concluded that the type of CEM did not affect the course of pH and conductivity development in the electrochemical cells, and the pH gradient could be successfully maintained between the electrolytes.

### 3.2. Assessment of Electrochemical Urea Stabilization

The kinetics of the stabilization process can be further evaluated by studying the extent of formation of OH^-^ ions (ΔOH^−^) necessary for achieving a targeted pH value. Presuming 100% Faradaic efficiency, ΔOH^−^ can be expressed according to Equation (4)

ΔOH^−^ = *I* · *t*/(*F* · *V_U_*) (4)

where *I* is the applied current (A), *t* is the operation time, *F* is the Faraday constant and *V_U_* is the volume of the catholyte, respectively.

**Figure 3:**
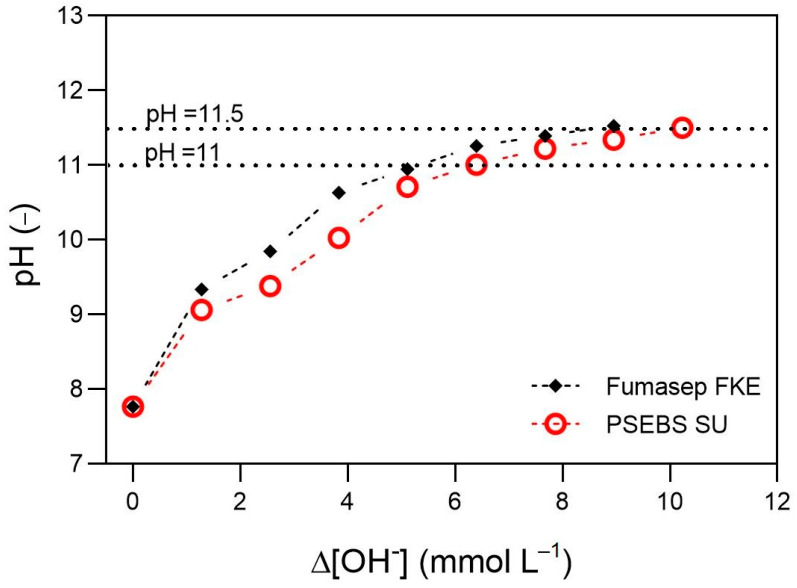
The change of catholyte pH as a function of produced hydroxyl ions.

Figure 3 shows the relationship between ΔOH^−^ and the catholyte pH. It can be seen that at the beginning of the process, the pH increases faster as the OH^−^ ions are produced, after which the curve gradually flattens. The Fumasep FKE-based cell showed a slightly more efficient process as less OH^−^ ions were able to ensure certain pH values during the operation. For example, in order to achieve pH = 11, a ΔOH^−^ of around 5.3 mmol OH^−^ L^−1^ was required, while it was nearly 6.4 mmol OH^−^ L^−1^ for the PSEBS SU-equipped cell. The differences between these values for the different CEMs may be attributed to the different ratios of positively charged species (Na^+^ and H^+^) migrating through the different membranes. Although presumably the transport of Na^+^ ions is predominant, the characteristics (mostly the combined effects of higher swelling and ionic conductivity [22]) of PSEBS SU may lead to increased H^+^ transport towards the catholyte, reacting with OH^−^ ions. However, this aspect is yet to be investigated in detail.

In order to increase the pH value to 11.5, an additional amount of 3.6 and 3.8 mmol OH^−^ L^−1^ was needed in the case of Fumasep FKE and PSEBS SU, respectively. This corresponds to 68% and 59% of ΔOH^−^ used for increasing the pH from the initial value of 7.77 to 11. This clearly indicates that the ΔOH^−^ required for conducting the stabilization process further from pH = 11.5 until reaching pH = 12 could be comparable to the ΔOH^−^ necessary for ensuring pH = 11.5. To estimate the effectiveness of the target value of pH = 11.5, the stability of the electrochemically treated solution should be evaluated. Urea stabilization should be efficient enough to ensure storage without urea loss until further processing. It was shown previously that increasing the pH to 11 is insufficient, as only short-term (1 week) stability could be achieved, while the pH = 12—although being significantly more time- and energy-consuming to achieve—allows stable storage over 18 months [15]. In this work, urea stability at an intermediate of 11.5 was investigated at room temperature (20–22 °C) and 4 °C.

**Figure 4:**
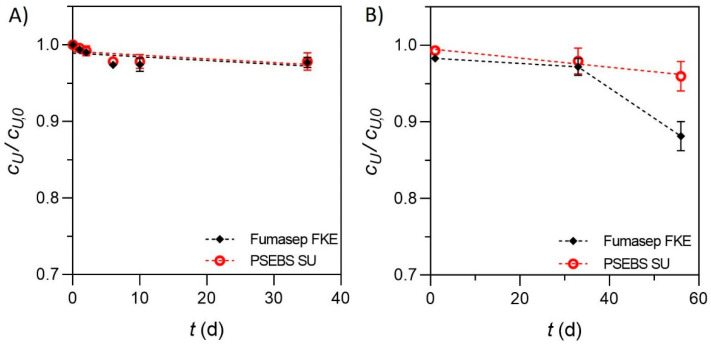
Urea stability evaluation over time (pH = 11.5) at room temperature (**A**) and 4 °C (**B**).

Comparing the actual (*c_U_*) and initial (*c_U,0_*) urea concentrations of stabilized solutions (stabilization efficiency), it could be observed that there was no significant urea decrease at room temperature within the 35 d long tests; thus, on average, 97.5 ± 0.8% of the initial amount of urea was detected in the solution, regardless of the membrane used (Figure 4A). As for the samples stored at 4 °C, on day 35, the results were nearly identical to the ones measured at room temperature, meaning a 97.5 ± 1.4% stabilization efficiency (Figure 4B). After 56 d, slight differences could be seen between the solutions obtained with different membranes. While the PSEBS SU-derived sample showed a more linear decrease in *c_U_*/*c_U,0_* (i.e., 95.9 ± 1.9%), the sample from the Fumasep FKE-based experiments showed a slightly higher loss of urea concentration, resulting in *c_U_*/*c_U,0_* = 88.1 ± 1.9% (Figure 4B).

It is important to mention that after 35 days, microbial contamination was present in the samples stored at room temperature, most probably due to sampling. Therefore, stability tests were not conducted further. Such phenomena were not experienced in the case of the samples stored at 4 °C. It can be said, based on the results, that urea can be sufficiently stabilized at pH = 11.5, allowing long-term and efficient storage. In general, a slight, ~2.5% per month decrease could be seen in the urea concentration of the stored samples. The higher decrease observed in the case of the Fumasep FKE-based solution may rather be attributed to either a sample handling issue (e.g., microbial contamination) or other analytics aspects, or to differences related to the CEM. This phenomenon, as well as the storability over longer time periods, should be further investigated.

In terms of the energy requirements of the stabilization process, the theoretical and measured electric energy consumption (*eec*) can be determined according to Equation (5),

*eec* = (*E_cell_* · *I* · *t*)/*V_U_* (5)

where *E_cell_* is the cell potential. For theoretical and measured *eec*, *E_cell_* is calculated from the theoretical anode and cathode potentials obtained from the Nernst equation at a given pH, or replaced by the measured cell voltage, respectively. In the presented experimental setup and conditions, the theoretical *eec* was 0.43 kWh m^−3^, considering an average cell potential of 1.55 V. However, the measured cell voltage was significantly higher during the operation, namely, 4.38 V, which resulted in a measured *eec* of 1.2 kWh m^−3^.

### 3.3. Efficiency of Simultaneous Phosphorous Recovery

One major advantage of the electrochemical stabilization is that due to the high pH value, the phosphate ions form precipitates with the Ca^2+^ and Mg^2+^ ions present in the catholyte. This phenomenon has two potential benefits. On the one hand, it allows for simultaneous and separate P-recovery from urine during urea stabilization. On the other hand, removing the Ca^2+^ and Mg^2+^ ions from the solution is an effective tool to counteract chemical scaling on the electrode’s (and ion exchange membrane’s) surface.

For the evaluation of phosphate recovery during the electrochemical stabilization process, the final P concentration of the catholyte was determined after gently removing the liquid phase from the settled precipitate. Based on the results, it could be concluded that compared to the initial phosphate concentration, the resulting P content decreased by up to 82.3 ± 2.8%, regardless the CEM used. Compared to the various electrochemical techniques shown in the related literature, the presented experimental approach and P-recovery can be considered as sufficient. For example, Cid et al. [26] achieved up to 80% P removal (mostly in the form of hydroxyapatite) by electrochemical cathodic precipitation using toilet wastewater under a 3–4 h operation time and a 50 A m^−2^ current density in a multi-anode multi-cathode system. In the work of Paepe et al. [15], a CEM-equipped electrochemical reactor was used to stabilize source-separated urine by pH modulation at 60 A m^−2^, which resulted in 34.7% P removal in the form of precipitate. Using anion exchange membranes (AEM), P removal could be enhanced; however, it turned out that the excess removal was mostly due to migration through the AEM.

**Figure 5:**
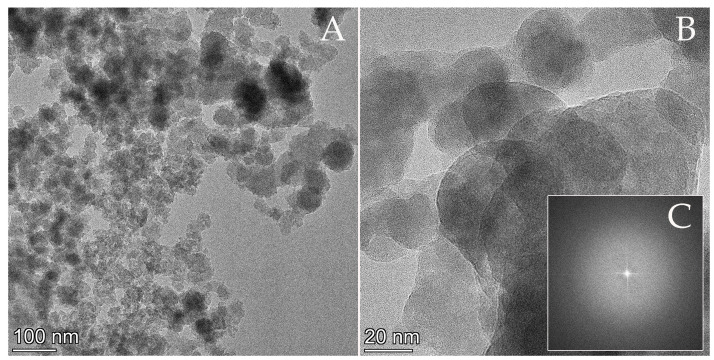
(**A**) BF and (**B**) HRTEM images of an aggregate of nanoparticles in the precipitate sample. The small inset image (**C**) is the Fourier transform of the HRTEM image (**B**), indicating that the particles have an amorphous structure.

The TEM study of the precipitate showed aggregates of nanometer-sized particles (Figure 5A). The size of the irregular particles varies within a narrow range between ~20 and 70 nm. The high-resolution (HRTEM) image of an agglomerate with associated fast Fourier transform (FFT) showed no lattice fringes or reflections that would be characteristic of crystalline material (Figure 5B,C).

**Figure 6:**
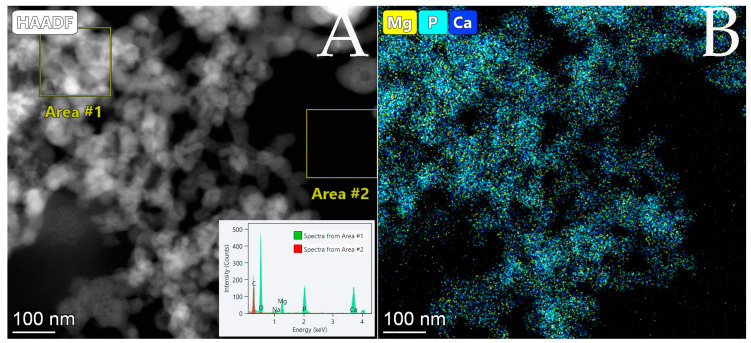
(**A**) STEM HAADF image with corresponding EDS elemental map (**B**), showing the uniform distribution of Mg, P and Ca, and extracted EDS spectrum from the areas marked 1 (the precipitate) and 2 (the background) showing the elemental composition of the sample.

Further elemental analyses of the amorphous particles were performed in scanning transmission mode (STEM). The EDS elemental map shows the distributions of P, Ca and Mg (Figure 6B) and the EDS spectrum shows the elemental composition (Figure 6A inset image) of the sample. Two areas were analyzed, the particles and the background, indicating that the carbon content mainly comes from the background carbon film. Mainly O, P, Ca and Mg were present in the particles, thus the flake-like precipitate could be identified as amorphous calcium magnesium phosphate (CMP).

Previous research undertaken with a similar experimental setup showed that the formed precipitates do not have a crystalline structure, and are potentially richer in calcium [15]. Our results are in good agreement with those suggestions and demonstrate the calcium-rich amorphous nature of the precipitate. The obtained CMP may have good potential as an efficient fertilizer for acidic soils, as it provides a good amount of plant-available phosphorous. Further changes in the amorphous precipitate (e.g., developing crystalline structure over time) could be studied.

## 4. Conclusions

The electrochemical pH modulation of synthetic urine for separate and simultaneous N and P recovery was carried out in an electrochemical cell setup equipped with different CEM. It was concluded that compared to a commercial reference CEM of Fumasep FKE, the developmental PSEBS SU CEM demonstrated sufficient performance, resulting in similar urine stabilization efficiency, and an average P recovery by cathodic precipitate (CMP) formation of >82% was achieved. The target pH of 11.5 could be attained in both systems after 4 h operation time at 60 A m^−2^ current density. This final pH was also sufficient for stabilizing the urine by inhibiting the enzymatic hydrolysis of urea. The pH-modulated solutions showed excellent stability during storage. The results presented here could provide a good basis for further process development and offering a deeper insight into the effects of various parameters on the stabilization and precipitation aspects. Future studies should focus on developing the electrochemical cell design, investigating the pH-dependency of the treated urea-rich solution’s stability, the effects of calcium and magnesium concentration on the precipitate formation and kinetics, and evaluating the actual fertilizer potential of the obtained CMP.

## Data Availability

Not applicable.

## References

[1] Kavvada O., Tarpeh W.A., Horvath A., Nelson K.L. (2017). Life-cycle cost and environmental assessment of decentralized nitrogen recovery using ion exchange from source-separated urine through spatial modeling. Environ. Sci. Technol..

[2] Liu Y., He L.F., Deng Y.Y., Zhang Q., Jiang G.M., Liu H. (2022). Recent progress on the recovery of valuable resources from source-separated urine on-site using electrochemical technologies: A review. Chem. Eng. J..

[3] Ledezma P., Kuntke P., Buisman C.J.N., Keller J., Freguia S. (2015). Source-separated urine opens golden opportunities for microbial electrochemical technologies. Trends Biotechnol..

[4] Mobley H.L.T., Hausinger R.P. (1989). Microbial ureases: Significance, regulation, and molecular characterization. Microbiol. Rev..

[5] Ray H., Saetta D., Boyer T.H. (2018). Characterization of urea hydrolysis in fresh human urine and inhibition by chemical addition. Environ. Sci. Water Res. Technol..

[6] Udert K.M., Larsen T.A., Gujer W. (2006). Fate of major compounds in source-separated urine. Water Sci. Technol..

[7] Larsen T.A., Udert K.M., Lienert J. (2015). Source Separation and Decentralization for Wastewater Management.

[8] Udert K.M., Larsen T.A., Biebow M., Gujer W. (2003). Urea hydrolysis and precipitation dynamics in a urine-collecting system. Water Res..

[9] Udert K.M., Larsen T.A., Gujer W. (2003). Estimating the precipitation potential in urine-collecting systems. Water Res..

[10] Larsen T.A., Riechmann M.E., Udert K.M. (2021). State of the art of urine treatment technologies: A critical review. Water Res. X.

[11] Arve P.H., Popat S.C. (2021). Stabilization of Urea for Recovery from Source-Separated Urine Using Electrochemically Synthesized Hydrogen Peroxide. ACS ES&T Eng..

[12] Arve P. (2022). Stabilization of Urea in Urine through the Electrochemical Generation of Hydrogen Peroxide. ECS Meet. Abstr..

[13] Xu L., Ding R., Mao Y., Peng S., Li Z., Zong Y., Wu D. (2021). Selective recovery of phosphorus and urea from fresh human urine using a liquid membrane chamber integrated flow-electrode electrochemical system. Water Res..

[14] Perera M.K., Englehardt J.D. (2020). Simultaneous nitrogen and phosphorus recovery from municipal wastewater by electrochemical pH modulation. Sep. Purif. Technol..

[15] De Paepe J., De Pryck L., Verliefde A.R.D., Rabaey K., Clauwaert P. (2020). Electrochemically Induced Precipitation Enables Fresh Urine Stabilization and Facilitates Source Separation. Environ. Sci. Technol..

[16] Govindan K., Im S.J., Muthuraj V., Jang A. (2021). Electrochemical recovery of H2 and nutrients (N, P) from synthetic source separate urine water. Chemosphere.

[17] Perera M.K., Englehardt J.D., Cohn J.L., Dauer E.A., Shukla D. (2020). Electrohydromodulation for phosphate recovery from wastewater. Sep. Purif. Technol..

[18] Hellström D., Johansson E., Grennberg K. (1999). Storage of human urine: Acidification as a method to inhibit decomposition of urea. Ecol. Eng..

[19] Zhang Y., Li Z., Zhao Y., Chen S., Mahmood I.B. (2013). Stabilization of source-separated human urine by chemical oxidation. Water Sci. Technol..

[20] Randall D.G., Naidoo V. (2018). Urine: The liquid gold of wastewater. J. Environ. Chem. Eng..

[21] Randall D.G., Krähenbühl M., Köpping I., Larsen T.A., Udert K.M. (2016). A novel approach for stabilizing fresh urine by calcium hydroxide addition. Water Res..

[22] Koók L., Rosa L.F.M., Harnisch F., Žitka J., Otmar M., Nemestóthy N., Bakonyi P., Kretzschmar J. (2022). Functional stability of novel homogeneous and heterogeneous cation exchange membranes for abiotic and microbial electrochemical technologies. J. Memb. Sci..

[23] Sarigul N., Korkmaz F., Kurultak İ. (2019). A New Artificial Urine Protocol to Better Imitate Human Urine. Sci. Rep..

[24] Hoseney R.C., Finney K.F. (1964). Spectrophotometric Determination of Urea, Thiourea, and Certain of Their Substitution Products with p-Dimethylaminobenzaldehyde and Diacetylmonoxime. Anal. Chem..

[25] Dick W.A., Tabatabai M.A. (1977). Determination of Orthophosphate in Aqueous Solutions Containing Labile Organic and Inorganic Phosphorus Compounds. J. Environ. Qual..

[26] Cid C.A., Jasper J.T., Hoffmann M.R. (2018). Phosphate Recovery from Human Waste via the Formation of Hydroxyapatite during Electrochemical Wastewater Treatment. ACS Sustain. Chem. Eng..

